# Effect of OLED Waste Glass Powder on Early Strength Performance of Rapid-Hardening Concrete

**DOI:** 10.3390/ma19051004

**Published:** 2026-03-05

**Authors:** Ngan Thanh Vu, Seong-Kyum Kim, Jae-Min Lee

**Affiliations:** 1 Department of Civil Engineering, Kumoh National Institute of Technology, Gumi 39177, Republic of Korea; 2 School of Architecture, Civil and Environmental Engineering, Kumoh National Institute of Technology, Gumi 39177, Republic of Korea; skim@kumoh.ac.kr

**Keywords:** OLED waste glass powder, rapid-hardening concrete, strength performance, early-ages

## Abstract

Rapid-hardening concrete is widely used for rapid repairs but can suffer from accelerated hydration, shrinkage-related cracking, and durability concerns. This study evaluates the feasibility of replacing cement with OLED waste glass powder (0–30%) in CSA-type rapid-hardening concrete as a low-impact repair material. Mixtures were prepared at a constant binder content (400 kg/m^3^) and water-to-binder ratio (0.425), and fresh properties (slump, air content, setting time) and mechanical performance (compressive and bond strength) were tested from 4 h to 56 d. Mercury intrusion porosimetry (MIP) and TG/DTG were additionally used to interpret changes in pore structure and hydration-related thermal indices. Increasing glass powder replacement improved workability but delayed setting. A 10% replacement (O-GP10) maintained 4 h compressive strength and showed slightly higher long-term strength and consistently higher long-term bond strength than the control, whereas 20–30% replacement caused pronounced strength loss due to dilution. MIP results indicated that O-GP10 suppressed large pores (>0.1 μm) and promoted a refined pore structure dominated by finer pores. TG/DTG trends were interpreted using temperature windows as comparative indicators, suggesting age-dependent bound-water development and a reduced apparent contribution in the Al-bearing-hydrate-related region for O-GP10. Overall, roughly 10% OLED waste glass powder is suggested for CSA rapid-hardening concrete to ensure early functioning while enhancing long-term bonding and microstructural stability.

## 1. Introduction

Bridge expansion joints, runways, or handling emergency pavement work are often subjected to deterioration, degradation, and damage by the natural environment during operational times [1,2,3]. These problems have resulted in significant mechanical and durability loss that requires rapid repair in order to extend lifecycle and save costs [4]. It is essential to not only resolve urgent works where the interruption was limited as far as possible, but also ensure the remaining early strength materials are suitable for the long term [5]. Rapid-hardening cement, known as calcium sulfoaluminate cement (CSA), which is commonly used for rapid repair, provides notable benefits in terms of time and high-performance mechanical qualities within a few hours [6]. Unlike ordinary Portland cement (OPC), CSA cement is highlighted as having high early strength and superior bonding strength [7]. Due to high alite contents, it actively reacts to promote hydration, and the CSH gel and CSA react to form needle-shaped ettringite crystals, which increase the strength in the early stages [8].

Notwithstanding the interest in rapid-hardening concrete (RHC) materials due to their potential to reduce construction time without compensating for the heat-curing cost for emergency repairs, their drawbacks in terms of environmental impacts and long-term performance remain unclear. Firstly, RHC material could enable fast setting and rapid hardening, leading to a greater demand for cement production compared to conventional concrete, and has been highlighted as one of the factors contributing to the increase in global CO_2_ emissions [9]. In particular, mixtures of these repair materials are commonly designed with a high binder content and low water-to-binder ratio to meet the early strength and fast-reopening requirements of infrastructure conditions [10]. However, such mixture characteristics often accelerate hydration heat and autogenous shrinkage, leading to early-age cracking and slower early-age strength development in pore structure. Therefore, these impacts could cause risks related to reduced mechanical performance and long-term durability, despite the benefits of initial strength [11]. The environmental impact of rapid-hardening repair materials is dominated by emissions from the binder production stage, and the cradle-to-gate global warming potential (GWP) of Portland cement is reported to be approximately 0.73–0.84 kg CO_2_ eq based on environmental product declarations [12]. In contrast, ground-glass pozzolan produced by grinding waste glass is mainly driven by electricity-based grinding processes, and EPDs report its cradle-to-gate GWP to be about 0.096–0.150 kg CO_2_e [13]. Therefore, partially replacing cement with supplementary cementitious materials (SCMs) such as recycled glass powder offers a quantitative pathway to reduce CO_2_e emissions at the material production stage.

In recent years, the incorporation of supplemental cementing materials (SCMs) into calcium sulfoaluminate (CSA) rapid-hardening cement has gained popularity as a realistic solution to the limits of rapid-hardening binders. Supplementary cementitious materials (SCM_*s*_) offer the advantage of lowering the risks of drying shrinkage, deformation, and cracking caused by initial heat generation, while also enhancing workability properties [9]. These alternative materials significantly minimize CO_2_ emissions and environmental consequences due to high binder requirements [10]. Some of these investigations, using the isothermal calorimetry model, explicitly stated that integrating SCMs such as fly ash, gypsum and limestone powder could effectively diminish or delay heat flux peaks and reduce the total heat released in calcium sulfoaluminate (CSA) mixtures, thereby offering the potential to mitigate early thermal-related damages while decreasing cement consumption [11,14,15].

Nevertheless, concerns persist that the partial substitution of calcium sulfoaluminate (CSA) with supplementary cementitious materials (SCMs) may compromise its fundamental characteristics, particularly the rapid setting and early strength development, which could significantly increase the impact on highway traffic disruption [16]. Earlier studies indicated that the inclusion of calcined coal gangue (CCG) reduces cement’s early strength due to the “dilution” effect and slower early response rate [17]. Additionally, its low early strength and delayed setting time considerably impede the usage of potential SCMs, especially when its replacement ratio exceeds a given threshold [18]. Findings indicate that the reduction in early strength is manageable when utilizing SCMs at moderate replacement levels and with sufficiently fine particles, which can sustain and enhance early strength. Scholars also suggest that the optimal balance between early-age performance and long-term strength enhancement is achieved with a 10–30% replacement of cement with fly ash [19]. This is attributed to a combinatorial mechanism, involving the physical effects of fillers and packing agents that optimize particle size distribution, decrease the interparticle space, facilitate rapid particle coalescence, and enhance nucleation points, thereby expediting hydrate deposition and formation from the initial hydration and kinetics (e.g., accelerated ettringite formation) in the CSA composition [20]. For example, low to medium fly ash replacement could promote CSA hydration via the filler effect, achieving equivalent or higher compressive strength than those achieved without replacing under controlled water/binder ratio conditions, and fine limestone powder can also accelerate the initial hydration reaction by providing seed sites [15,21,22]. Simultaneously, up-to-date studies on the addition of C–S–H seeds sites demonstrate that the acceleration due to nucleation impacts and rapid pozzolanic reaction could produce C–S–H gel and compensate for the initial strength degradation often associated with clinker reduction. In addition, C–S–H nano-seed sites have been shown to possess a synergistic effect in enhancing the initial mechanical properties in CSA-containing binders [23,24].

From this point of view, OLED waste glass powder is generally considered to have alkali-free properties like glass in the display industry and can be produced as a fine amorphous silica-based powder; thus, it can be expected to not only have pozzolanic reactivity but also filler/nucleation effects and is evaluated as a promising candidate for an SCM that can relatively reduce ASR problems, which are a major issue in waste soda-lime glass [25,26,27].

As a result, if treated OLED glass powder (grinding, particle size control, etc.) is used at an appropriate substitution rate, it is expected that the “early strength enhancement and rapid setting” properties of the CSA repair material will be maintained, while ensuring the benefits of reduced hydration heat and total CO_2_ emissions by cement usage demands. Nonetheless, this is an approach that has not been fully developed for CSA-based rapid-hardening concrete for repair works.

As discussed earlier, this study examines the feasibility of partially replacing activated OLED glass powder (O-GP) in CSA-based rapid-hardening concrete to mitigate the heat of hydration at early ages and to ensure the potential for reducing CO_2_ emissions solutions through using replacing pozzolanic material without compromising the rapid-hardening and early strength performance that is required for emergency repair works.

In the experimental design, the O-GP substitution ratio with a fixed w/b of 0.425 was determined as the only major factor, and the effectiveness of the hydration reaction at each substitution ratio was quantitatively compared using mixtures of substitution levels of 0%, 10%, 20%, and 30%. In addition, fresh concrete was evaluated through slump, air content, and setting time tests, which were used to comprehensively assess workability and the potential for reopening within a short time. Furthermore, mechanical performance was determined using compressive strength and bond strength tests, allowing for evaluation of the usefulness of O-GP in both early-age development and long-term performance. Additionally, in order to underscore the microstructural development and changes in the hydration reaction process due to the substitution of O-GP content based on four levels (0, 10, 20, 30), analysis of pore structure (MIP) and thermogravimetric analysis (TGA) were performed. Along with this, the formation and changes in hydration products were compared and discussed in this study. This indicates the contributions of O-GP glass powder to RHC properties, and through this, the effect of O-GP partial replacement on the performance mechanism (workability–compressive strength–microstructure) of CSA rapid-hardening concrete used in repair materials has been investigated, offering a fundamental experimental basis for the mix design of sustainable rapid-hardening repair materials.

## 2. Materials and Experimental Methods

### 2.1. Raw Material

**Figure 1:**
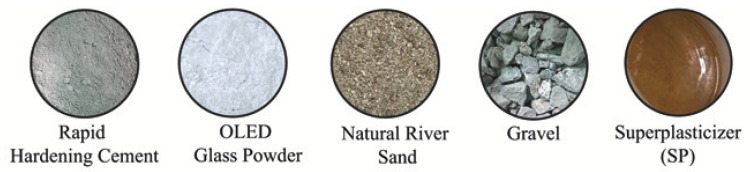
Raw materials.

Figure 1 depicts the raw materials in this study, which were utilized as binders, including CSA-type rapid-hardening cement (RHC) manufactured by Union Cement Co., Ltd. (Seoul, Republic of Korea), OLED waste glass powder supplied by LG Display Co., Ltd. (Gumi, Republic of Korea), river sand as fine aggregates, gravel provided by Geumo aggregate company (Gumi, Republic of Korea) and a polycarboxylate-based high-performance water-reducing agent (PCE) manufactured by Dongnam Co., Ltd. (Seongnam, Republic of Korea). Table 1 summarizes the X-ray fluorescence (XRF)-based oxide composition and important physical properties of the two materials. RHC was dominated by CaO (42.85%), with high levels of Al_2_O_3_ (12.84%) and SO_3_ (13.44%). In particular, CSA-based rapid-hardening cements often contain high levels of Al_2_O_3_ and SO_3_, which promotes the formation of ettringite products during the initial hydration process. This creates a favorable material foundation for rapid setting and early strength development.

The average particle size of RHC was measured to be 30.5 μm, the specific gravity was 2.89 g/cm^3^, and the specific surface area was 5760 cm^2^/g, indicating a relatively high level of fineness. The loss on ignition was confirmed to be 2.8%. Meanwhile, the OLED glass powder was primarily composed of SiO_2_ (61.6%) and also exhibited a relatively high level of Al_2_O_3_ (19.7%). Conversely, CaO content was low at 4.6%, demonstrating its distinct characteristics as a Si–Al-based glass (aluminosilicate) precursor. Furthermore, it contained oxides typically observed in display substrate glass, such as BaO (7.85%), SrO (1.78%), and B_2_O_3_ (1.64%), demonstrating compositional characteristics distinct from those of typical soda-lime glass powder. The average particle size of the OLED glass powder was 35.1 μm, similar to that of RHC, in the micrometer range, but its specific gravity was lower at 2.49 g/cm^3^. The specific surface area was measured to be 2635 cm^2^/g.

**Figure 2:**
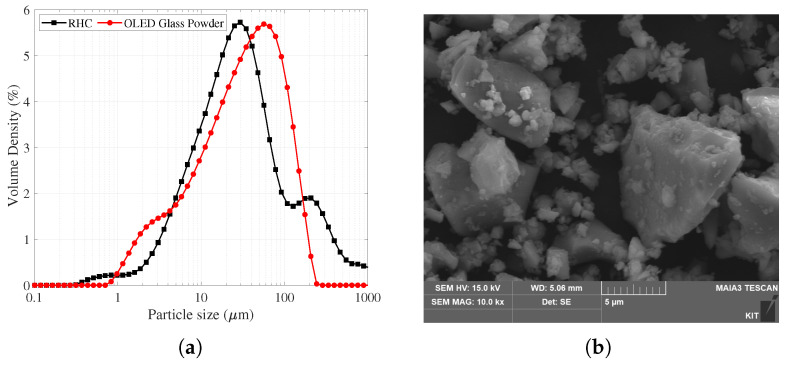
Particle size distribution of RHC and OLED glass powder (**a**) and SEM micrograph of OLED glass powder (**b**).

Figure 2 presents the particle size distribution (PSD) of the binder. Both powders show a wide distribution range from several μm to several hundred μm, and the main volumetric distribution tends to be concentrated in the range of about 10–150 μm. Comparing the curve shapes, the OLED glass powder (red) shows a rightward shift in the center of distribution toward relatively large particle sizes. On the other hand, the cement-based powder (black) shows a high-volume density peak in the medium particle size range, but a relatively long tail in the coarse particle region. This suggests that the OLED glass powder has a relatively narrow particle size distribution, while the cement-based powder may still have some contribution from the coarse particle population due to crushing, agglomeration, and a mixture of particle sizes. Consequently, the binder system in this study can be interpreted as a mixed binder in which (i) the high fineness of RHC and early reactivity based on the sulfoaluminate-based composition; and (ii) the filling effect and potential contribution to long-term reactivity due to the Si–Al-based glassy composition and particle size characteristics of the OLED glass powder work together.

**Figure 3:**
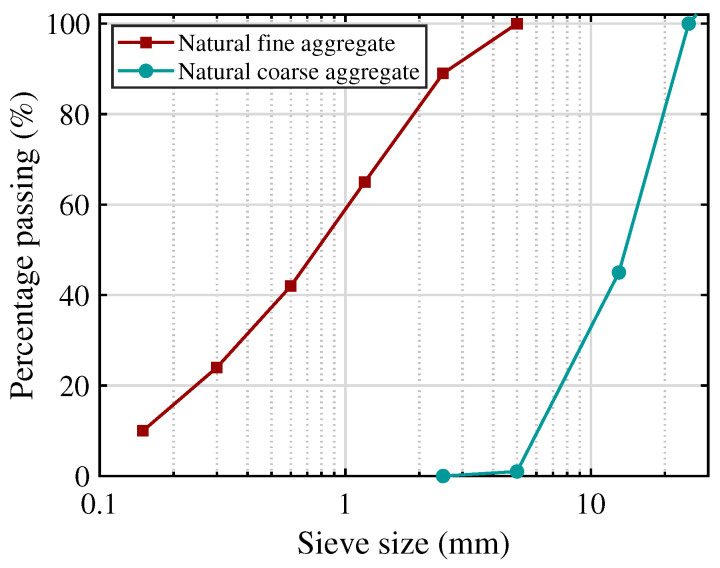
Grading curves of aggregates.

In this study, natural river sand was used as fine aggregate and crushed stone was used as coarse aggregate. According to Table 2, the specific gravity of fine aggregate and coarse aggregate was measured as 2.55 and 2.63 g/cm^3^, the absorption rate was 0.91% and 1.07%, and the soundness loss was 1.5% and 2.4%, respectively. The 0.08 mm sieve passage rate was confirmed to be 3.7% for fine aggregate and 0.4% for coarse aggregate, indicating that the fines content of coarse aggregate was very low, and the fine aggregate also did not contain excessive fines. Figure 3 shows the particle size curves of fine and coarse aggregates. In addition, a polycarboxylate-based high-performance water-reducing agent (PCE) with a water reduction rate of 21% was used as an admixture. The water-reducing agent was pre-dissolved in the mixing water and then added during the mixing process, and municipal tap water was used as the mixing water.

### 2.2. Experimental Methods

#### 2.2.1. Mix Proportions and Preparation

The mixtures were prepared to assess how the substitution of cement with OLED waste glass powder affects the early strength performance of concrete. Four mixes were used (O-GP0, O-GP10, O-GP20, and O-GP30), including a control mix (O-GP0). In these mixtures, a portion of the cement in the binder was progressively substituted with OLED glass powder, as summarized in Table 3. The water-to-binder ratio (W/B) was maintained at 0.425 for all mixes, and the total binder content was fixed at 400 $\mathrm{kg}/m^{3}$. The OLED glass powder substitution rate was varied to 0%, 10%, 20%, and 30% (by mass). The mixtures were prepared using a 50 L pan-type forced mixer. The dry materials were first mixed for 2 min to ensure uniform distribution. Afterward, the pre-measured mixing water containing the dissolved superplasticizer was added, and wet mixing was continued for an additional 3 min. The fresh concrete was cast into molds in two layers. Each layer was compacted using a vibration table for 30 s to eliminate entrapped air and ensure proper consolidation. After casting, the specimens were demolded 4 h later and subsequently cured in water at a temperature of 20 ± 2 °C until the specified testing ages.

To clearly compare the effects of OLED glass powder substitution, the aggregate conditions were the same across all mixes. The coarse and fine aggregate contents were fixed at 1140 $\mathrm{kg}/m^{3}$ and 637 $\mathrm{kg}/m^{3}$, respectively. Additionally, to ensure the workability, a polycarboxylate-based high-performance water-reducing agent (superplasticizer (SP)) was applied equally to all mixtures at 1.0% (4 $\mathrm{kg}/m^{3}$) based on the mass of the binder.

#### 2.2.2. Fresh Properties

To analyze the characteristics of fresh concrete, slump, air content, and setting time were measured immediately after mixing. Slump and air content were measured using rapid-hardening concrete, while setting time was measured using rapid-hardening mortar, from which coarse aggregate was removed to eliminate interference from the penetration resistance. Slump, air content, and setting time were tested according to ASTM C143 [28], ASTM C231 [29], and ASTM C807 [30], respectively.

#### 2.2.3. Mechanical Properties

**Figure 4:**
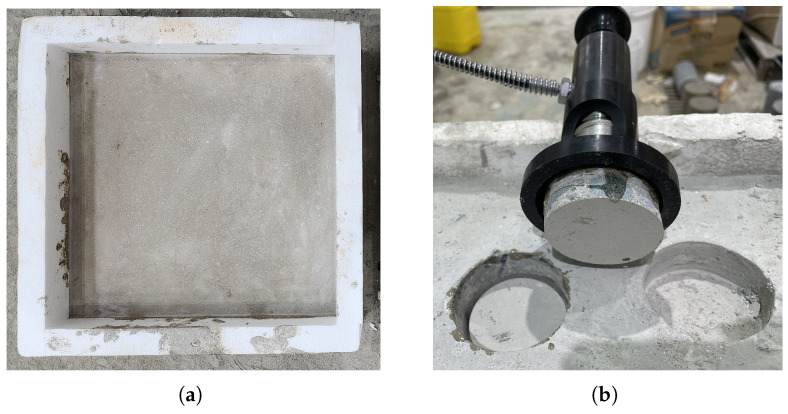
Pull-off test specimen (**a**) before and (**b**) after failure, respectively.

**Figure 5:**
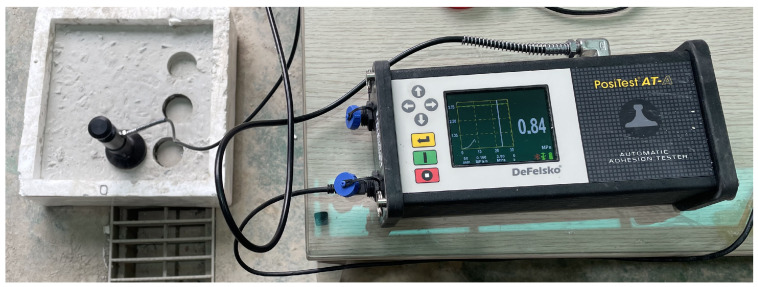
Pull-off testing instrument.

The mechanical behavior of rapid-hardening concrete incorporating OLED powder was examined using compressive strength and interfacial bond strength tests. Measurements were taken after curing periods of 4 h, 1 d, 14 d, 28 d, and 56 d. Compressive strength was determined at each age after water immersion curing, following the procedures specified in ASTM C39 [31]. Cylindrical specimens with dimensions of Ø100 × 200 mm were used to measure the compressive strength of each mixture. A universal testing machine with a maximum loading capacity of 2000 kN was employed, and the loading rate was maintained at 118 kN/min. Interfacial bond strength was assessed in accordance with ASTM C1583 [32] to evaluate the bonding performance between the existing hardened concrete substrate and the newly added concrete. The substrate specimens were first prepared and cured for 28 days, as shown in Figure 4a. After the concrete substrate block was surface-treated according to standard procedures, the concrete under study was poured over it to form composite specimens of the new concrete layer and the existing one. To measure the bond strength, a 50 mm diameter circular metal disk was adhesively attached to the surface of new concrete, as shown in Figure 5. After the adhesive hardened, a core was drilled in a circular pattern around the outer perimeter of the disk to ensure that the tensile load was transferred from the disk to new concrete, the interface, and the base concrete. Afterwards, a vertical tensile load was applied to the disk using a dedicated pull-off instrument to measure the maximum load at the time of failure, as shown in Figure 4b. The bond strength was calculated as the nominal tensile stress by dividing the maximum failure load by the cross-sectional area of the disk.

#### 2.2.4. Pore Structure Analysis

Mercury Intrusion Porosimetry (MIP) was performed to quantitatively analyze the micropore structure. Pore measurements were determined with a pressure range of 0–113 MPa, and the mercury properties for data interpretation were set to a surface tension of 0.485 N/m and a contact angle of 130°.

Test specimens were selected from fragments generated after compressive strength testing (cylindrical specimens). To minimize the influence of external surface, 2–3 mm sized fragments were collected from the inner (core) region. Also, to terminate the hydration reaction and eliminate any remaining moisture, the collected fragments were immersed in ethanol and then dried at 50 °C for 4 days before MIP measurements were performed.

In order to minimize the influence of compressive loading on the pore structure analysis, MIP fragments were collected from the inner (core) region of the broken specimens, and fragments near visible fracture surfaces were intentionally avoided. Nevertheless, it should be acknowledged that compressive loading may introduce additional microcracks, which could partially influence the measured pore size distribution, particularly in the larger pore range. Since the same sampling procedure was consistently applied to all mixtures, the comparative trends between specimens remain meaningful.

#### 2.2.5. Thermogravimetry (TG/DTG)

To quantitatively assess the amount of C–S–H gel formation based on CH (portlandite) consumption, thermogravimetric analysis was performed. To eliminate the influence of aggregate and potential interpretational interference, paste samples were prepared separately without aggregate. After reaching a predetermined age, the samples were immersed in isopropanol for 24 h to halt the hydration reaction. This samples were then vacuum-dried at 50 °C and ground to a particle size of less than 100 μm to prepare powder samples for analysis. Thermogravimetric analysis (TGA) was performed in a nitrogen atmosphere over a temperature range of 20–1000 °C, with a heating rate of 10 °C/min. CH consumption was assessed by calculating the mass loss during the CH decomposition region from the TG/DTG curve, and the characteristics of hydration product (C–S–H gel) formation were compared and analyzed based on this data.

## 3. Results and Discussion

### 3.1. Influence of OLED Waste Glass Powder Content on the Fresh Properties of Rapid-Hardening Concrete

#### 3.1.1. Slump Test

**Figure 6:**
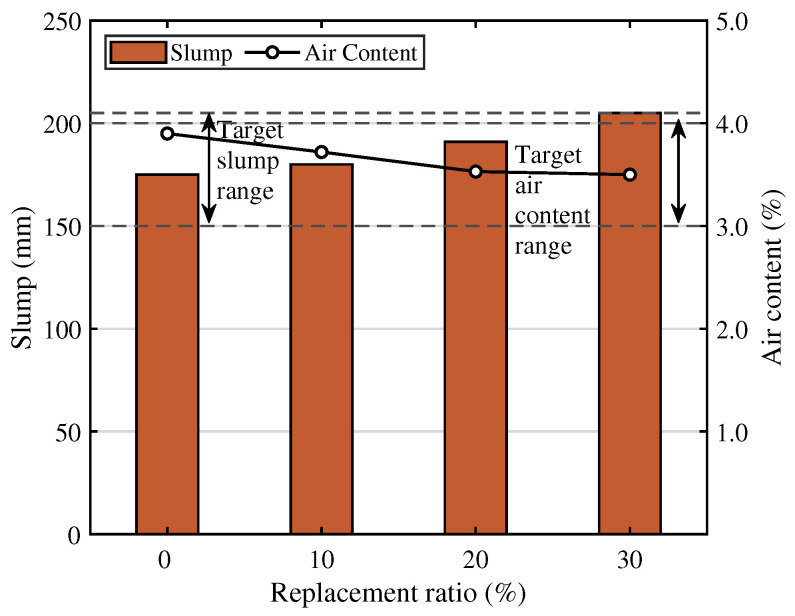
The slump and air content test results.

The slump and air content test results for OLED rapid-hardening concrete are presented in Figure 6. As the replacement ratio of OLED waste glass powder increased, the mixture fluidity gradually increased. This result turns out to be closely related to the physical properties of the OLED waste glass powder. In this study, the average particle size of the OLED waste glass powder used was approximately 35 μm, similar to the average particle size of the rapid-hardening cement used (roughly 30 μm). However, the specific surface area of the OLED waste glass powder and cement was significantly different: 2635 cm^2^/g the O-GP and 5760 cm^2^/g for the RHC. This suggests that, based on the same mass, this OLED waste glass powder has a relatively lower surface area than the cement, potentially reducing the amount of binding water required during mixing.

Under these circumstances, a glass powder with a lower specific surface area requires less water to wet the powder surfaces using the same unit water quantity, resulting in an increase in free water (effective water content). This free water increase is regarded as a key element that may accelerate the fluidity of concrete by inducing behavior similar to an increase in the effective water-to-binder ratio [33,34,35]. The non-absorbent property and relatively smooth particle surface of glass powder are also crucial factors. Compared to porous mineral admixtures (e.g., some fly ash, GGBS, metakaolin), OLED glass has a very low water absorption rate, and its relatively smooth particle surface tends to reduce frictional resistance between particles within the mixture [36,37,38]. This lubricating effect can be particularly pronounced as the replacement ratio increases. A study on self-compacting concrete (SCC) reported that slump flow tends to increase with increasing replacement ratio of crushed OLED waste glass [39]. This is primarily attributed to the combined effect of glass powder acting as a microfiller, improving interparticle gaps and simultaneously reducing internal friction.

#### 3.1.2. Air Content

The air content results presented in Figure 6 show a gradual drop, from 3.90%, to 3.72%, 3.53%, and 3.50%, as the OLED waste glass powder replacement ratio increases. The mechanism here aligns with the behavior observed in the slump results: it comes down to free water content and surface characteristics. Unlike RHC, OLED waste glass powder is non-absorbent with a lower specific surface area, meaning less water gets trapped, which reduces the “bound water” on the powder surface under the same unit water content conditions. Consequently, the volume of free water effectively rises [33,34,35,40]. Physically, the glass’s smoother texture plays a critical role by minimizing interparticle friction, which directly improves the mixture’s fluidity [38,39,40]. With this improved fluidity, the mixture can more effectively purge entrapped air during the pouring and compaction processes, thereby slightly reducing the total air content [41,42]. Specifically, researchers have noted that replacing cement with glass powder yields a tighter air content range than OPC, particularly when the design mix is tuned for particle size [35]. A similar mechanism is used in foundational studies on OLED waste aggregates. There, the reduction is attributed to surface texture: the inherent smoothness of the glass prevents air voids from stabilizing at the paste–aggregate interface, causing a noticeable drop in air content as replacement levels rise [42].

In summary, the investigation of both increased slump and decreased air content with increasing OLED waste glass powder replacement ratio can be attributed to the physical properties of OLED waste glass powder since it has a lower specific surface area (2635 cm^2^/g vs. 5760 cm^2^/g) compared to OPC, and possesses a non-absorbent, smooth surface. By liberating more free water and lowering the friction between particles, this physical characteristic directly boosts the mixture’s fluidity. Since the workability improves, the mix becomes more efficient at expelling entrapped air during the mixing and casting phases.

#### 3.1.3. Setting Time

The results of the setting time (initial and final) test of OLED rapid-hardening concrete are presented in Table 4. The experimental test was conducted using the method of measuring concrete setting by penetration resistance, and it was revealed that both the initial and final setting times tended to be delayed as the replacement ratio of OLED waste glass powder increased. In particular, the initial setting time was 28.95 min with a replacement ratio of 0%. This upward trend continued as the replacement levels increased. The setting time extended to 29.17 min at 10% and reached 31.53 min at the 20% mixtures, respectively. This result was most pronounced at the 30% level, where the setting time climbed to 35.50 min. This underscores a clear gap of roughly 6.55 min between the 30% mixture and the 0% control.

A similar delay also appeared in the final setting times, which stretched from 32.45 min in the control mixture to 33.61 min at 10%, 37.36 min at 20%, and 40.56 min at 30% dosages. By the 30% replacement ratio, the initial setting was delayed by around 8.11 min relative to the RHC control mix. The main reason for this retardation is due to the altered initial hydration environment created by the physical and chemical properties of the replacing OLED waste glass powder.

Firstly, the most immediate influence is the binder’s diluting effect: less cement means less reactive clinker minerals to accelerate the hydration process. Simultaneously, the presence of glass powder appears to hinder the early hydration stage, thereby delaying the formation of the interparticle bridges (C–S–H and ettringite) required for early setting [43]. These findings are consistent with the current literature, which presents that initial and final setting times frequently increase as the replacement ratio rises. Since glass powder exhibits negligible pozzolanic activity in the early stages, it effectively dilutes the system without contributing to the initial set, resulting in a delayed setting process in the early hydration stages [44,45]. In addition, as discussed in the slump and air content results of this study, OLED waste glass powder has a lower specific surface area and a surface characteristic close to non-absorbent compared to cement. Therefore, it might enhance the free water content of the entire mixture while maintaining the same unit water content. Finally, it should be noted that increasing the water–cement ratio (or water–binder ratio) or decreasing the fineness of cement powder also leads to delayed setting time [46].

In this study, the experimental investigation reported that replacing glass powder led to an increase in the effective water–binder ratio. Simultaneously, the amount of reactive clinker per unit volume decreased, which likely delayed the formation of initial hydration products and pushed back the initial setting time of the rapid-hardening system. Consequently, the observed settling delay with increasing OLED waste glass powder replacement can be attributed to the combined effects of the increased free water content in the mixture, which delays the formation of initial interparticle networks, and the glass powder’s reactive contribution, which primarily occurs at later ages. This property can be advantageously utilized in terms of initial working time when designing eco-friendly binders using OLED waste glass powder [44,45,46].

### 3.2. Influence of OLED Waste Glass Powder Content on Mechanical Performance

#### 3.2.1. Compressive Strength

**Figure 7:**
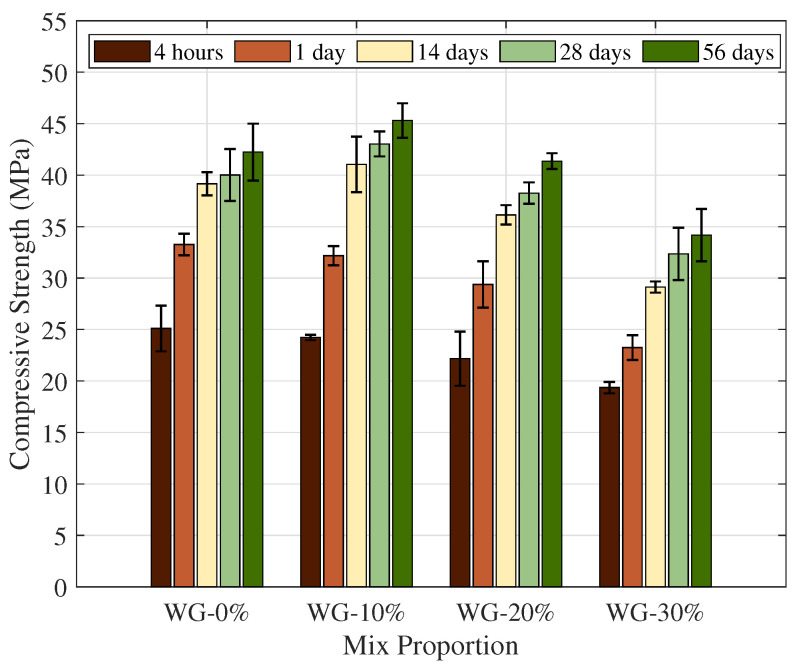
Influence of OLED waste glass powder on the compressive strength of RHC specimens.

**Figure 8:**
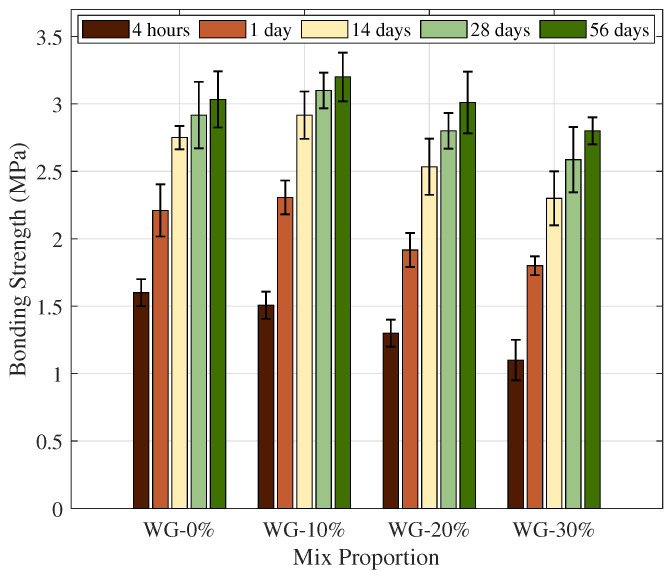
Influence of OLED waste glass powder on the bonding strength of RHC specimens.

Figure 7 and Figure 8 depict the evolution of compressive and bond strengths across the dosage range of 0%, 10%, 20% and 30%. As a characteristic of this CSA binder system, the early-age performance is underscored by a sharp surge in strength within the first 24 h. This rapid acquisition of strength is mainly attributed to the rapid formation of ettringite (AFt) during the primary hydration reaction and its high initial reactivity. This has been widely discussed in the field of rapid repair [47,48].

In Figure 7, the control (O-GP0) specimens demonstrated consistent strength gain over time: starting from 25.1 MPa at 4 h, the strength rose to 33.2 MPa (1 day), 39.1 MPa (14 days), and 40.0 MPa (28 days), before finally stabilizing at 42.2 MPa by day 56. This is consistent with previous reports that CSA-based rapid-hardening cements rapidly develop strength at early ages [47,48]. Furthermore, the relatively gradual strength increase from 14 d onwards is consistent with the high initial reactivity of CSA-based materials, as well as the previous reports that the rate of strength increase in the longer term can be slowed down by micro-cracking accumulated by autogenous shrinkage and thermal gradients in the early stages [49].

O-GP10 had an early strength of 24.2 MPa at 4 h, which was similar to the control group (25.1 Mpa), and after 56 days, it remained slightly stronger at 45.3 MPa than the control group (42.2 MPa). This minor strength loss at low replacement ratios is typically attributed to the fact that fine-powder admixtures induce strength degradation due to cement dilution (dilution effect) and simultaneously provide a filler/nucleation effect that stimulates early hydration through particle filling and nucleation, thereby compensating for the early strength decline [47,50]. It has been found that initial performance degradation may not be considerable within a specific range of substitution ratios, even in CSA-SCM (e.g., fly ash) blends. The dilution effect becomes dominant as the replacement ratio increases [50].

Conversely, O-GP20 and O-GP30 showed considerable strength deterioration across all ages. O-GP20 depicted early strength loss of 22.1 MPa at 4 h and 29.3 MPa at 1 d, with a gap of 41.3 MPa at 56 days compared to the control. O-GP30 had the lowest results, 19.3 MPa at 4 h and 34.1 MPa at 56 days. These results can be summarized as a “dilution-dominant” behavior, where the absolute amount of CSA clinker (e.g., ye’elimite) decreases with increasing replacement ratio, limiting AFt formation and the total amount of hydration products, resulting in lower strength growth [47,50]. Glass powders are typically noted for their pozzolanic reactivity over extended durations; however, the intensity and timing of this reaction are considerably affected by variables such as binder composition, pore solution chemistry, and particle size. In numerous instances, even with a contribution from the reaction, it remains challenging to adequately offset clinker loss under high-exchange conditions [27,51,52].

#### 3.2.2. Bonding Strength

The bond strength of a repair material is commonly regarded as being more prone to be affected by the quality of the substrate–repair interface and the failure mode, such as pure interface failure, repair material internal failure, partial substrate failure, or substratum failures [53,54,55]. Also, determining the bonding strength requires more than just a fundamental compressive strength measurement.

In Figure 8, the control group (O-GP0) increased from 1.6 MPa at 4 h to 3.0 MPa after 56 days. CSA-based repair materials have been depicted to achieve early bond strength through fast-setting and early strength development. Early bonding strength is a crucial factor in determining the usefulness of rapid repair materials [48,56]. O-GP10 maintained a similar level to the control group (1.6 MPa) at 1.5 MPa at 4 h; however, after 1 day, it moderately surpassed the control group at 2.3 MPa (control group 2.2 MPa), 3.1 MPa (control group 2.9 MPa) at 28 days, and 3.2 MPa (control group 3.0 MPa) at 56 days. This suggests that although O-GP10 has lower total compressive strength than the control group at early ages, it offers superior performance corresponding to bonding strength in the long term. This compressive strength–bonding strength ratio corroborates existing research showing that the pore structure of the overlay transition zone (OTZ), the moisture state of the substrate, and the hydration and shrinkage stress of the interface during construction, as well as the accumulation of micro-cracks, considerably influence the bonding strength [32,54,55]. In particular, Beushausen et al. stated [54] that modifications in the moisture state of the underlying substrate and the OTZ porosity could significantly affect the bonding performance. Moreover, crack/contraction deformation is suggested as a major factor limiting the development of bonding strength [55,56]. In addition, G. Ke et al. [57] indicated that CSA concrete can be concentrated with contraction deformation at the very beginning of aging (immediately after setting); thus, it can be hypothesized that the replacement of O-GP10 may partially suppress the damage to the interface (incipient cracking or interfacial delamination) by easing the heat of hydration and deformation behavior.

O-GP20’s initial bond strength declined to 1.3 MPa at 4 h and 1.9 MPa at 1 day; however, it increased to 3.0 MPa at 56 days, corresponding to the control sample. This is in agreement with the global model for bond strength development in repair materials, and higher replacement ratios minimize initial reactivity, resulting in lower initial contact resistance at the bonding interface. Nonetheless, the evolution of interfacial hydration and mechanical interlockings happens when the curing age increases. This makes it possible to rehabilitate to a specific extent [32,56].

O-GP30 samples possess the lowest bonding strength throughout all curing ages (1.1 MPa at 4 h, 2.8 MPa at 56 d). The loss in strength is attributable to increased replacement rate, which limits the interfacial stress transfer capacity (tensile and interfacial resistance of the repair material) [32,50,56].

Overall, a low replacement (10%) proportion of OLED waste glass powder can minimize initial strength loss through filler/nucleation effects while mitigating interfacial damage (microcracks and OTZ degradation), which can be beneficial for long-term bond strength. However, at higher replacement ratios of 20–30%, dilution effects become dominant, resulting in overall deterioration in compressive and bond strength performance [27,50,56,57].

### 3.3. Influence of OLED Waste Glass Powder Content on Microstructural Development

#### 3.3.1. Pore Structure

This study examined the mechanical performance (compressive strength and bond strength) of mix proportions with replacement ratios of 0%, 10%, 20%, and 30%. As can be seen, O-GP0 and O-GP10 samples were found to be the only mix proportions that concurrently met the mechanical performance requirements for rapid-hardening concrete adhered to KS F 2762 [58] and KS F 2405 [59] (4 h bond strength ≥ 1.4 MPa, 28 d compressive strength ≥ 30 MPa, and 4 h compressive strength ≥ 21 MPa). As a result, to elucidate the pore structure of the representative mixes selected for their practical feasibility, MIP-based microstructural analysis was conducted exclusively on the 0% and 10% replacement groups (O-GP0, O-GP10).

**Figure 9:**
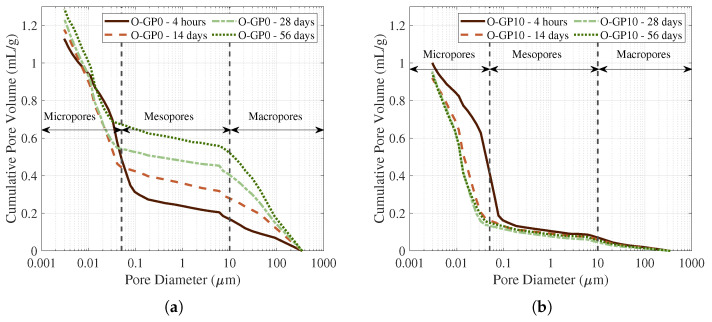
Cumulative pore volumes of O-GP0 (**a**) and O-GP10 (**b**) specimens.

**Figure 10:**
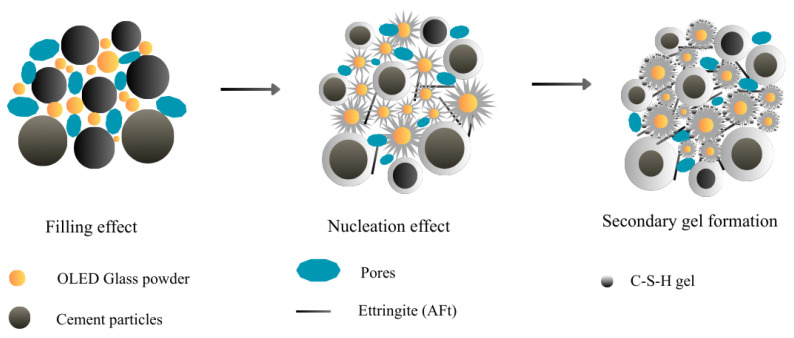
Schematic mechanism diagram of OLED glass powder into rapid-hardening concrete.

Figure 9 depicts how cumulative pore volume varies with pore size. The cumulative pore capacity of O-GP10 was notably large at 4 h (about 1.0 mL/g); however, it only subsequently remained modest (roughly 0.93–0.95 mL/g) throughout the following 14 to 56 days. Specifically, the volume of pores exceeding 0.1 μm decreased substantially. This densification aligns with the typical evolution of the paste: hydration products gradually occupy the space and lead to pore refinement, thereby reducing the presence of large voids and limiting the overall interconnectivity of the system [60,61]. In addition, there are many reports that attribute the long-term microstructural improvements to the synergistic effects of the fine admixture. Physically, the glass powder reduces porosity by filling initial voids and accelerating nucleation, as shown in Figure 10. Chemically, it modifies the hydration products by supplying reactive glassy Si, which generates additional C-(A)-S-H phases and progressively refines the pore system as the concrete matures [62].

**Figure 11:**
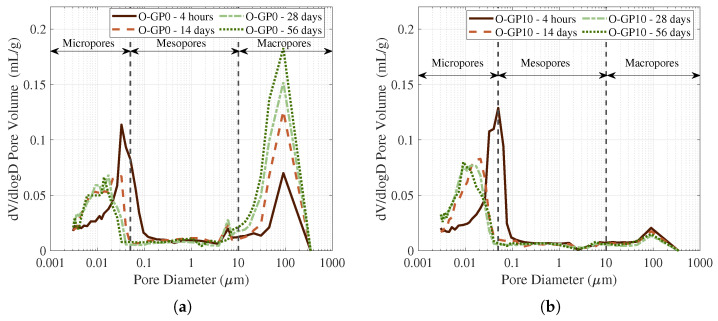
Differential pore size distribution of O-GP0 (**a**) and O-GP10 (**b**) specimens.

Figure 11 demonstrates the differential pore distribution based on dV/dlogD. Taken together, the pore distribution exhibits relatively distinct peaks or distribution changes in the micropore group (0.01–0.05 μm) and the macropore group at 10 μm or more. In O-GP10, with increasing aging, the macropore peak of several 10 μm or more is generally suppressed, and the micropore range (<0.05 μm) becomes more dominant, indicating a refined pore structure. This is consistent with the existing mechanism that pore refinement can be achieved through the filling and nucleation effects of glass powder and the formation of additional products through long-term reaction [62].

In contrast, the O-GP0 sample exhibits a micropore peak around 0.03–0.05 μm at 4 h, followed by a significant growth of a large peak in the tens of μm range at long ages. This evolution is indicative of progressive defect development in the pore network. Considering that CSA-based systems can be susceptible to volumetric instability at early ages, such defect growth may be related, at least in part, to shrinkage-driven microstructural disturbance [49]. In particular, when large pores (or defective paths) increase, the microstructure may be reorganized in a direction unfavorable to both strength and transport characteristics, specifically permeability and diffusion [61]. It should also be noted that the use of fragments obtained after compressive strength testing may partly contribute to the apparent increase in the large-pore fraction. Therefore, the observed increase in larger pores in the O-GP0 specimen at later ages is interpreted primarily in terms of microstructural deterioration mechanisms reported for CSA systems (e.g., shrinkage-related damage), while acknowledging that compressive loading could partially influence the measured results.

**Figure 12:**
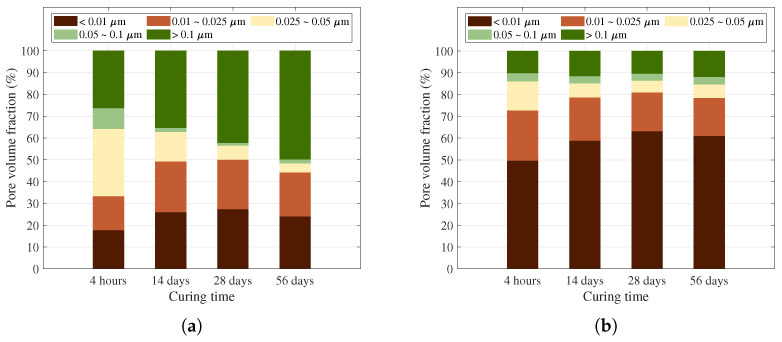
Pore volume fractions of O-GP0 (**a**) and O-GP10 (**b**) specimens.

Figure 12 compares the pore volume fractions across five pore size ranges: <0.01 μm, 0.01–0.025 μm, 0.025–0.05 μm, 0.05–0.1 μm, and >0.1 μm. The most striking difference is observed in the >0.1 μm range. In O-GP0, the >0.1 μm proportion increases from approximately 26% to 35% to 43% to 50% with increasing aging. In contrast, in the O-GP10 sample, the same range remains at approximately 10–12%, demonstrating a significantly lower contribution from large pores. These results underscore that the formation and connectivity of large voids are suppressed in O-GP10, and the void system is maintained in a smaller split state, which in turn can be interpreted as a favorable microstructure from the perspective of mobility characteristics [61,62]. In addition, the proportion of <0.01 μm (ultra-fine pores) is approximately 50–63% for O-GP10, which is significantly higher than that of O-GP0 (approximately 18–27%). On the other hand, it can be concluded that 10% glass powder replacement acts to rearrange the pore structure (pore refinement) toward a distribution centered on ultra-fine pores while suppressing large pores [62].

In summary, a direct association exists between pore structure reduction and strength performance increases [3]. As the microstructure densifies by decreasing the fraction of big pores and subdividing pore structure more finely, durability and strength capacity might be greatly increased. In this investigation, O-GP10 pointed out this tendency, defined by a clear shift from macropores (>0.1 μm) to the ultrafine range (<0.01 μm) relative to the O-GP0 control. This specific reconfiguration likely established a robust microstructural foundation favorable for maintaining and enhancing mechanical properties as the curing age increased [61,62]. Furthermore, bond strength is significantly influenced not only by the strength of the repair material itself but also by the microstructure of the overlay transition zone (OTZ), the moisture content of the substrate, interfacial void formation, and microcrack accumulation. In particular, it has been quantitatively reported that substrate moisture conditions directly influence the OTZ void ratio and connective tissue formation, supporting the view that densification of the interfacial pore structure is crucial for bond performance [54]. From this perspective, O-GP10 met the initial performance criteria (compression and bonding) at 4 h and slightly outperformed that of the control group over the long term due to the reduced risk of interface damage resulting from the inhibition of large pores and the organization of the pore system [54,62].

#### 3.3.2. Thermogravimetric Analysis (TGA)

In this study, TG/DTG analysis was performed at ages of 4 h, 14 d, 28 d, and 56 d on the CSA-based super-speed binder paste to evaluate the effect of 10% replacement (O-GP10) of OLED waste glass powder on hydration products and thermal decomposition behavior. In addition, TG/DTG analyses were performed on representative formulations (O-GP0 and O-GP10) that simultaneously satisfied both early and long-term performance criteria in the previous mechanical performance evaluation, thereby interpreting the results of the thermal decomposition behavior analysis consistently with the rationale used for the microstructure (MIP) analysis, allowing for a direct correlation between pore structure and hydration products. In thermal analysis, the 80–200 °C range is commonly interpreted as dehydration of AFt (ettringite) and bound water, while acknowledging that, in CSA-based binders, multiple hydrates may contribute to this low-temperature interval; 250–350 °C as dehydration of amorphous aluminum hydroxide (AH_3_) series, which is herein treated as an Al-bearing-hydrate-related interval (including AH3-like contributions) due to possible overlap among aluminum-containing hydrates in CSA systems; approximately 400–500 °C as dehydration of portlandite (CH), and 700–800 °C as decarbonation of carbonates (mainly CaCO_3_). This segmentation approach is universally adopted in allocating typical TG/DTG peaks and the quantitative assessment of cement-based hydrates [63,64,65], but the window-based values in CSA systems should be interpreted primarily as comparative indicators rather than exclusive quantification of single phases.

**Figure 13:**
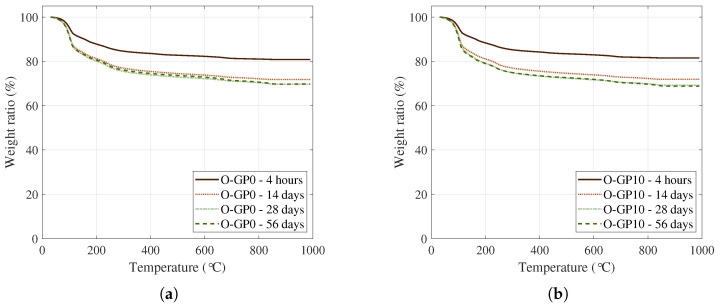
Thermogravimetric analysis of O-GP0 (**a**) and O-GP10 (**b**) specimens.

**Figure 14:**
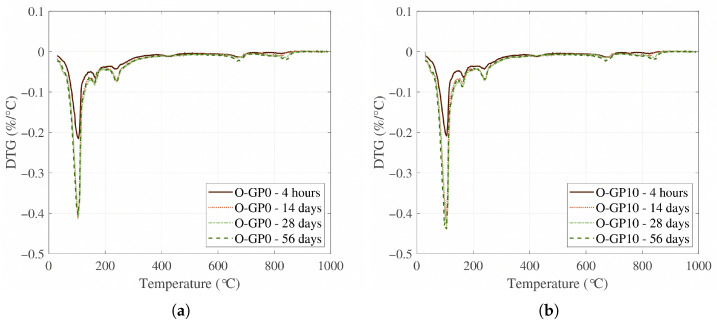
Differential thermogravimetric analysis of O-GP0 (**a**) and O-GP10 (**b**) specimens.

**Figure 15:**
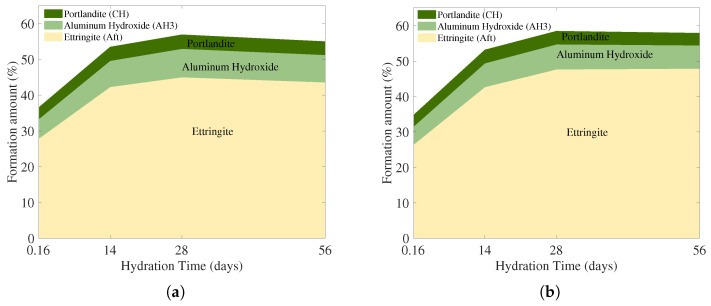
Formation amount of O-GP0 (**a**) and O-GP10 (**b**) specimens.

In the TG and DTG results (Figure 13 and Figure 14), both O-GP0 and O-GP10 gradually developed weight loss and corresponding DTG peaks in the 80–200 °C range as the aging increased. This is aligned with the general trend of increasing bound water components due to AFt (ettringite) and calcium silicate aluminate hydrate gel (C-(A)-S-H, etc.) [66,67,68], which are typically formed in CSA-based binders, as shown in Figure 15. In particular, O-GP10 showed a lower overall weight reduction contribution and DTG peak in the 250–350 °C range (the Al-bearing-hydrate-related interval, often associated with AH_3_-like dehydration) compared to O-GP0. This suggests that the OLED waste glass powder replacement modifies the hydration pathway: specifically, it lowers the relative proportion of the 250–350 °C Al-bearing-hydrate-related contribution and reorganizes available aluminous components into more gel-phase (C-(A)-S-H/C-A-S-H) or other aluminous-containing hydration products [66,69,70]. Attributing these microstructural changes solely to a ’simple dilution’ effect is insufficient. A more plausible explanation incorporates the active contribution of pozzolanic reaction or latent hydration reaction, in which the free waste glass powder dissolves (releases) Si species from a high-alkali aqueous solution and reacts with Ca and Al over time to induce the formation of secondary hydration gel (C-(A)-S-H/C-A-S-H) [71,72,73].

On the other hand, for the region near 400–500 °C (the region commonly assigned to CH dehydroxylation), the signals remained stable in both mixtures, with no clear upward trend observed. Unlike OPC, CSA-based binders do not accumulate CH in a predominant (even if formed, its relative proportion remained low) manner, and the interpretive sensitivity of this fraction may increase depending on the mixture reaction and carbonation history [69,70,74].

In this study, the hydrate content was estimated using a temperature-window approach, which converts the weight loss within a specific temperature range of the TG curve (Figure 13) [67,75]. However, in CSA-based binders, multiple hydrates, particularly Al-bearing phase, may dehydrate over overlapping temperature intervals. Therefore, rather than being an absolute quantification, it is appropriate to use these data as relative markers of apparent content, mainly for comparing the evolution between mixes and curing ages. Based on this rationale, O-GP10 consistently exhibited lower apparent Al-bearing-hydrate-related (250–350 °C) indices than O-GP0 across all ages. In contrast, AFt/hydrogel-related components, typically represented in the 80–200 °C range, tended to accumulate steadily with increasing age. This suggests that 10% replacement of OLED waste glass powder maintained the initial AFt-based hydration structure in CSA hydration, while altering the hydration phase composition over time by redistributing some of the aluminous components to gel phases or other aluminous-containing hydration phases rather than independently accumulating as a single, uniquely quantifiable “AH_3_” phase. Consequently, the relative prominence of the 250–350 °C Al-bearing-hydrate-related contribution is reduced [66,69,70].

Notably, the characteristics of O-GP10 can be explained by connecting them with the pozzolanic reaction as follows. The glassy powder may have limited reactivity at the beginning (several hours to several days), but with prolonged aging and Si dissolution, Ca-Al-Si-based hydrogels are further formed, densifying the microstructure, which can be reflected in thermal analysis as an increase in the bound water content in the range of 80–200 °C [71,72,73]. Furthermore, it has been widely discussed that pozzolanic reactions are not limited to simple CH-consuming reactions but can also change the formation pathway of C-(A)-S-H/C-A-S-H and related Al-silicate hydrated phases (e.g., strätlingite series) by combining with Ca-sources in the system (such as a Ca-containing phase and a dissolved Ca^2+^ generated in the hydration process) [67,72,76]. Therefore, the simultaneous reduction in the 250–350 °C Al-bearing-hydrate-related index and the robust presence of AFt/Gel components in O-GP10 reflect the synergistic role of the OLED glass powder replacement, as shown in Table 5. These attributes are due to the mechanism where the material first aids hydration products through the fine powder effect (packing and nucleation), then drives the system toward organizing the hydrated phase combination around the gel/AFt center by supplying glassy silica as necessary as the aging progresses for secondary gel formation [71,72,73].

## 4. Conclusions

This study evaluates the effects of replacing OLED waste glass powder with cement in CSA-type rapid-hardening concrete using replacement ratios (0–30%). The objective is to ascertain the optimal replacement range while maintaining the water-to-binder ratio (0.425) and binder content (400 kg/m^3^) at a constant level in all mix proportions. Fresh properties and mechanical performance (4 h–56 d) were evaluated, and the underlying mechanisms were interpreted through pore structure (MIP) and hydration-related thermal indices (TG/DTG). Increasing the OLED glass powder replacement improved workability, as evidenced by higher slump and lower air content, which is attributed to the non-absorbent nature of the glass particles and the associated increase in available free water. However, higher replacement levels led to delayed initial and final setting, reflecting the combined effects of increased free water and early-age dilution with limited initial reactivity of the glass powder. Performance outcomes clearly depended on the replacement level. At 10% replacement (O-GP10), compressive strength was maintained at 4 h and slightly increased at later ages, whereas 20–30% replacement caused a consistent strength reduction across all ages, indicating dilution-dominated behavior at high replacement levels. Bond strength exhibited greater interfacial sensitivity than compressive strength: O-GP10 matched the control at early age and exceeded it at later ages, suggesting enhanced long-term interfacial performance and a reduced risk of damage accumulation at the repair interface. Microstructural analysis also supported these trends. MIP results showed that O-GP10 suppressed the contribution of large pores (>0.1 μm) and promoted a refined pore structure dominated by finer pores, consistent with improved long-term performance. TG/DTG results indicated progressive development of the low-temperature bound-water-related region (80–200 °C) with age. In CSA systems, dehydration signals of Al-bearing hydrates may overlap; therefore, the temperature-window approach was used as a comparative indicator rather than an absolute quantification of single phases. Within this framework, O-GP10 exhibited a lower apparent contribution in the 250–350 °C Al-bearing-hydrate-related region, together with sustained accumulation in the bound-water/gel-related region, which is consistent with the proposed mechanism of pore refinement driven by fine powder effects (filling and nucleation) and subsequent gel formation associated with glass dissolution and longer-term reactions. Overall, a 10% replacement of OLED waste glass powder (O-GP10) is optimal for performance, maintaining strength and bonding in rapid-hardening structures. However, once the replacement level exceeds 20%, the dilution effect takes over, causing a marked drop in both early and long-term strength. Therefore, this study proposes limiting the dosage to approximately 10%, which is the optimal level for repair projects requiring immediate traffic restoration without compromising durability.

## Figures and Tables

**Table 1:** Chemical and physical properties of RHC and OLED glass powder.

Properties		RHC	OLED Glass Powder
Oxide composition (wt.%)	CaO	42.85	4.60
	SiO_2_	13.72	61.60
	Al_2_O_3_	12.84	19.70
	Fe_2_O_3_	3.51	0.01
	MgO	2.29	2.50
	SO_3_	13.44	–
	K_2_O	0.65	0.01
	Na_2_O	–	0.03
	SnO_2_	–	0.29
	BaO	–	7.85
	SrO	–	1.78
	B_2_O_3_	–	1.64
Mean particle size (μm)		30.50	35.10
Specific gravity (g/cm^3^)		2.89	2.49
Specific surface area (cm^2^/g)		5760	2635
Loss on ignition (%)		2.80	0.70

**Table 2:** Properties of fine and coarse aggregates.

Properties	Fine Aggregate	Coarse Aggregate
Specific gravity (g/cm^3^)	2.55	2.63
Water absorption (%)	0.91	1.07
Soundness loss (%)	1.5	2.4
Material passing 0.08 mm sieve (%)	3.7	0.4

**Table 3:** Mix proportions of RHC and OLED glass powder specimens ($\mathrm{kg}/m^{3}$).

Mix Proportions (kg/m^3^)							
Specimens	W/B	Cement	OLED Waste Glass Powder	Water	Fine Aggregate	Coarse Aggregate	Superplasticizers (SP)
O-GP0	0.425	400	0	170	637	1140	4
O-GP10		360	40				
O-GP20		320	80				
O-GP30		280	120				

**Table 4:** Setting time OLED rapid-hardening concrete specimens.

Sample	Initial Setting Time (min)	Final Setting Time (min)
O-GP0 (RHC)	28.95	32.45
O-GP10	29.17	33.61
O-GP20	31.53	37.36
O-GP30	35.5	40.56

**Table 5:** O-GP0 and O-GP10 formation amount.

	O-GP0				O-GP10			
Phase Window	4 h	14 d	28 d	56 d	4 h	14 d	28 d	56 d
Aft	27.97	42.29	45.01	43.58	26.55	42.68	47.67	47.88
${\mathrm{AH}}_{3}$	5.46	7.31	7.95	7.65	5.12	6.71	7.06	6.56
CH	3.27	3.89	3.96	3.78	3.26	3.81	3.76	3.48

## Data Availability

The original contributions presented in this study are included in the article. Further inquiries can be directed to the corresponding author.
